# Plasma Lysyl-tRNA Synthetase 1 (KARS1) as a Novel Diagnostic and Monitoring Biomarker for Colorectal Cancer

**DOI:** 10.3390/jcm9020533

**Published:** 2020-02-15

**Authors:** Ji Hun Suh, Min Chul Park, Peter C. Goughnour, Byung Soh Min, Sang Bum Kim, Woo Yong Lee, Yong Beom Cho, Jae Hee Cheon, Kang Young Lee, Do-Hyun Nam, Sunghoon Kim

**Affiliations:** 1 Medicinal Bioconvergence Research Center, College of Pharmacy, Seoul National University, Seoul 08826, Korea; svhi2002@snu.ac.kr (J.H.S.); minchul.park@neomics.com (M.C.P.); petergoughnour@yahoo.com (P.C.G.); saintgene@biocon.snu.ac.kr (S.B.K.); 2 Department of Molecular Medicine and Biopharmaceutical Sciences, Graduate School of Convergence Technology, Seoul National University, Seoul 08826, Korea; 3 Seoul Republic of Korea Department of Surgery, Severance Hospital, Yonsei University College of Medicine, Seoul 03722, Korea; bsmin@yuhs.ac (B.S.M.); kylee117@yuhs.ac (K.Y.L.); 4 Department of Surgery, Samsung Medical Center, Sungkyunkwan University School of Medicine, Seoul 06351, Korea; wooyong123.lee@samsung.com (W.Y.L.); yongbeom.cho@samsung.com (Y.B.C.); 5 Department of Health Sciences and Technology, Samsung Advanced Institute for Health Sciences and Technology, Sungkyunkwan University, Seoul 06351, Korea; nsnam@skku.edu; 6 Department of Internal Medicine and Institute of Gastroenterology, Yonsei University College of Medicine, Seoul 03722, Korea; geniushee@yuhs.ac; 7 Institute for Refractory Cancer Research, Samsung Medical Center, Seoul 06351, Korea; 8 Department of Neurosurgery, Samsung Medical Center, Sungkyunkwan University School of Medicine, Seoul 06351, Korea

**Keywords:** lysyl-tRNA synthetase, colorectal cancer, serologic biomarker, diagnosis, monitoring, noninvasive method

## Abstract

Colorectal cancer (CRC) is one of the leading causes of world cancer deaths. To improve the survival rate of CRC, diagnosis and post-operative monitoring is necessary. Currently, biomarkers are used for CRC diagnosis and prognosis. However, these biomarkers have limitations of specificity and sensitivity. Levels of plasma lysyl-tRNA synthetase (KARS1), which was reported to be secreted from colon cancer cells by stimuli, along with other secreted aminoacyl-tRNA synthetases (ARSs), were analyzed in CRC and compared with the currently used biomarkers. The KARS1 levels of CRC patients (*n* = 164) plasma were shown to be higher than those of healthy volunteers (*n* = 32). The diagnostic values of plasma KARS1 were also evaluated by receiving operating characteristic (ROC) curve. Compared with other biomarkers and ARSs, KARS1 showed the best diagnostic value for CRC. The cancer specificity and burden correlation of plasma KARS1 level were validated using azoxymethane (AOM)/dextran sodium sulfate (DSS) model, and paired pre- and post-surgery CRC patient plasma. In the AOM/DSS model, the plasma level of KARS1 showed high correlation with number of polyps, but not for inflammation. Using paired pre- and post-surgery CRC plasma samples (*n* = 60), the plasma level of KARS1 was significantly decreased in post-surgery samples. Based on these evidence, KARS1, a surrogate biomarker reflecting CRC burden, can be used as a novel diagnostic and post-operative monitoring biomarker for CRC.

## 1. Introduction

Colorectal cancer (CRC) is highly prevalent and is the second leading cause of cancer-associated deaths in the world [1,2]. The five-year survival rate of CRC is approximately 50% and the incidence of CRC is increasing [3]. The survival rate of CRC is related with metastasis and recurrence. When colorectal cancer is properly diagnosed in the early stage or recurrence is detected by post-operation monitoring, it can be cured through surgery and/or drug treatment increasing the survival rate. For these reasons, an adequate diagnosis method is needed to improve the survival rate of CRC [4]. To date, several diagnostic tools have been developed to provide useful information on CRC patients [5]. The main diagnostic tests for CRC are stool-based tests or structural examinations such as endoscopy or radiographic imaging [6]. However, these tests have limited specificity, sensitivity, and convenience. For example, stool tests, including fecal occult blood testing (FOBT) and the fecal immunochemical test (FIT), might give false-positive results [7]. Although structural examinations, including colonoscopy and computer tomography, are effective methods with high sensitivity and specificity, they are expensive and cumbersome [8]. Therefore, more accurate and convenient diagnostic methods are needed.

To overcome these disadvantages, liquid biopsy and noninvasive and patient-compliant methods are emerging as alternative diagnostic strategies for CRC [9]. For precise liquid biopsy, suitable biomarkers should be developed and clinically validated. However, there is a lack of reliable and specific biomarkers for the diagnosis and prognosis of CRC [10]. For example, carcinoembryonic antigen (CEA), used as a blood marker for CRC diagnosis and monitoring [11], has limitations such as non-specificity. As CEA is also detected in many other types cancer, such as breast, lung, and pancreatic cancer and other malignancies [12], other diagnostic methods are required to confirm the CEA results. To overcome the non-sensitivity of biomarkers, a panel of biomarkers was analyzed for CRC diagnosis [13,14]. Although the panel biomarkers showed high sensitivity and specificity for diagnosis, due to non-association with clinicopathologic features, specific single biomarkers are needed for improved panel analysis [15]. For example, currently, methylated septin 9 (SEPT9), a Food and Drug Administration (FDA)-approved biomarker [16,17,18], is not only used as single biomarker, but also as panel analysis for higher specific and sensitive diagnosis.

Aminoacyl-tRNA synthetases (ARSs), which charge the amino acid to their cognate tRNA, are essential components of the protein translational machinery. In addition to their catalytic role, various non-canonical intracellular and extracellular biological activities of ARSs have been reported [19,20]. Recently, the extracellular activities of ARSs, secreted by different types of cells, were reported and validated in clinical samples [21]. Among the ARSs, unique behaviors of lysyl-tRNA synthetase 1 (KARS1) has been strongly associated with cancer. Whereas membrane KARS1 has been found to regulate tumor migration, secreted KARS1 exhibits proinflammatory functions in the cancer microenvironment [22,23]. The overexpressed membrane-bound KARS1 induces tumor cell migration for the progression of colon cancer [24]. KARS1 is normally anchored to the multi-synthetase complex (MSC) in the cytosol but released from MSC through caspase 8-mediated N-terminal truncation. Truncated KARS1 interacts with the syntenin–syndecan complex and is secreted via exosomes from cancer cells [25]. In colon cancer cells, KARS1 is secreted due to inflammatory stimuli [23]. Although the secretory pathway and function of KARS1 have been reported, its clinical relevance has not been validated yet. Overexpressed membrane-bound KARS1 and secreted KARS1 induce tumor cell migration and tumorigenic inflammation, respectively, for colon cancer progression [24]. Based on this, we analyzed the plasma levels of KARS1 in human CRC samples and in a colitis-induced CRC mouse model. In this study, the analysis of plasma KARS1 provided novel insights into the clinical relevance of KARS1 in CRC.

## 2. Materials and Methods

### 2.1. Collection of Human Samples

We collected 196 plasma samples from consecutively enrolled colorectal cancer (CRC) patients (*n* = 164) and healthy volunteers (*n* = 32) at the Samsung Medical Center, Institute for Refractory Cancer Research (SMC-IRCR, Seoul, Korea). Sixty pairs of plasma samples from pre- and post-surgery colorectal patients were obtained from Severance Hospital of Yonsei University (Seoul, Korea). The plasma collection procedure was approved by the Institutional Review Board (IRB) at SMC-IRCR (IRB No.: 2010-04-004) and Severance Hospital of Yonsei University (IRB No.: 4-2016-0955).

### 2.2. Materials

To make the stock solution, 10 mg of azoxymethane (AOM, Sigma, St. Louis, MO, USA) was dissolved in 1 mL of phosphate-buffered saline (PBS, Wellgene, Gyeongsan, Korea). This was diluted in isotonic saline (CJ, Seoul, Korea) to a concentration of 1 mg/mL for treatment. Further, 12.5 g of dextran sulfate sodium (DSS, MP Biomedicals, Illkirch-Graffenstaden, France) was dissolved in 500 mL of autoclaved water, passed through a 0.45-μm filter (Pall Corporation, New York, NY, USA), and stored at 4 °C for one week.

### 2.3. Inflammatory-Induced Colorectal Colitis Cancer Model

Mouse experiments were performed according to the Animal Care and Use Committee guidelines of Woojung Bio. Six-week-old male C57/BL6 mice (Nara Biotech, Seoul, Korea) were injected with 10 mg/kg of azoxymethane (AOM) or phosphate buffered saline (PBS) intraperitoneally (IP). They were given 250 mL of autoclaved water or 2.5% dextran sulfate sodium (DSS) every two days for one week. After one week, 2.5% DSS was replaced with autoclaved water for two more weeks, and this cycle was repeated three times. Every three weeks, the blood of mice was collected and stored at −80 ˚C. In the final week of the experiment, the mice were euthanized, and their colons were collected for analysis. The weights of the mice were measured on a weekly basis.

### 2.4. Harvesting Mouse Plasma Samples

The mice were anesthetized, and their blood was collected using the Retro-Orbital plexus method with a heparinized capillary tube (Kimble Chase, Rockwood, TN, USA). The samples were maintained at room temperature with heparin for 30 min for clotting and then centrifuged for 15 min at 2000× *g* in a pre-cooled centrifuge at 4 °C (Eppendorf, Hamburg, Germany). The supernatant, consisting of the plasma, was collected and used for assay. The plasma was harvested every three weeks.

### 2.5. Enzyme-Linked Immunosorbent Assay (ELISA)

Plasma biomarkers levels were determined using commercial ELISA kits according the manufacturer’s instructions. The samples from healthy controls and cancer patients were diluted at ratios of 1:3 and 1:10, respectively. The ELISA kits for tumor necrosis factor-α (TNF-α), interleukin 10 (IL-10) and interleukin 6 (IL-6) were purchased from BD Bioscience (San Diego, CA, USA), kits for glycyl-tRNA synthetase 1 (GARS1), histidyl-tRNA synthetase 1 (HARS1), and tryptophanyl-tRNA synthetase 1 (WARS1) were from Cusabio (Wuhan, China). The ELISA kits for aminoacyl tRNA synthetase-interacting multifunctional protein 1 (AIMP1) and CEA were from Elabscience (Wuhan, China), the KARS1 ELISA kit was from Mybiosource (San Diego, CA, USA), and the CA 19-9 ELISA kit was from Abnova (Taipei, Taiwan).

### 2.6. Statistical Analysis

Statistical analysis was performed using unpaired, two-tailed, Mann–Whitney U test for nonparametric groups: Healthy individuals and colorectal cancer patients. The plasma biomarker levels were indicated using a box (25th–75th percentile)-whisker (10th–90th percentile) plot. Using the results of ELISA, receiver operating characteristic (ROC) was performed to evaluate the discrimination properties between the healthy individuals and CRC patients as negative and positive, respectively. The area under the ROC curve (AUC) was used to evaluate the diagnostic accuracy of each marker. The cutoff value was calculated to maximize the total value of sensitivity and specificity computed from ROC analysis. The Pearson correlation coefficient (Pearson r) was used to analyze the correlation between tumor size and each biomarker level in the CRC patient plasma. The paired student’s t-test (two-tailed) was applied to compare the plasma biomarker levels in pre-/post-surgery CRC samples. ROC analysis was also performed using paired pre- and post-surgery CRC patient samples, and the sensitivity and specificity was calculated as previously described. The scattering dot plot was used to represent the number of polyps for the in vivo model. The *p* values were calculated using the Mann–Whitney test for the in vivo model. The statistical analysis was conducted using GraphPad Prism 8.3.0 and SPSS 25.0. The data were presented as mean ± SEM. *p* <0.05 was considered statistically significant.

## 3. Results

### 3.1. Plasma KARS1 Levels Were Increased in CRC Patients

**Figure 1:**
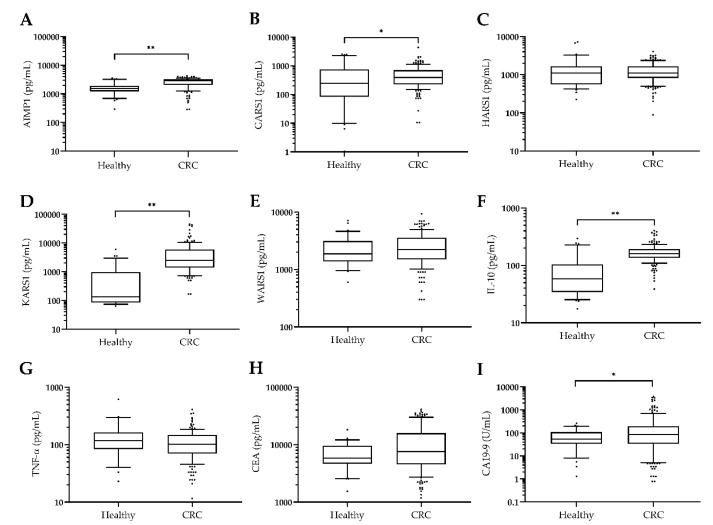
Levels of plasma biomarkers in colorectal cancer (CRC) patients and healthy controls. Levels: (**A**) Aminoacyl-tRNA synthetase-interacting multifunctional Protein 1 (AIMP1), (**B**) glycyl-tRNA synthetase 1 (GARS1), (**C**) histidyl-tRNA synthetase 1 (HARS1), (**D**) lysyl-tRNA synthetase 1 (KARS1), (**E**) tryptophanyl-tRNA synthetase 1 (WARS1), (**F**) interleukin 10 (IL-10), (**G**) tumor necrosis factor-α (TNF-α), (**H**) carcinoembryonic antigen (CEA), and (**I**) carbohydrate antigen (CA) 19-9 in the plasma of healthy control (*n* = 32) and CRC patients (*n* = 164) were graphed as box and whisker plots. Except for CA 19-9 (U/mL), pg/mL was used as the unit. *p*-Values were calculated using the Mann–Whitney U test. * *p* < 0.05, ** *p* < 0.0001.

To check the clinical relevance of plasma protein levels in CRC, blood samples were collected from 164 CRC patients and 32 healthy controls (the clinical characteristics are summarized in Table 1). To determine the plasma protein levels, ELISA was performed targeting secreted ARSs (AIMP1, GARS1, HARS1, KARS1, and WARS1) [26], cancer-associated cytokines (TNF-α and IL-10), and cancer biomarkers (CEA and CA19-9) (Figure 1). The plasma AIMP1, KARS1, and IL-10 levels were significantly higher in the colorectal cancer patients than the healthy controls (*p* < 0.0001). Although GARS1 showed a meaningful difference, the level of CRC patients was not higher than level of the healthy control group (*p* < 0.05). The median plasma levels of AIMP1, KARS1, and IL-10 were 2600 pg/mL, 5007 pg/mL, and 167.9 pg/mL in CRC patients, respectively and 1711 pg/mL, 775.6 pg/mL, and 87.4 pg/mL in the healthy control group, respectively (Table S1). However, there was no significant difference in the levels of known inflammatory cancer biomarkers, TNF-α and CEA, between the two groups (CEA: *p =* 0.0676, TNF-α: *p* = 0.196). Secreted ARS proteins, HARS1 and WARS1, in inflammation disease, did not show significantly difference between the two groups (HARS1: *p* = 0.602, WARS1: *p* = 0.2304) [21,27]. There was an appreciable difference in the plasma CA 19-9 levels, a well-known cancer biomarker, between the two groups (*p <* 0.05).

### 3.2. Plasma KARS1 Level Showed Diagnostic Potential in CRC

**Figure 2:**
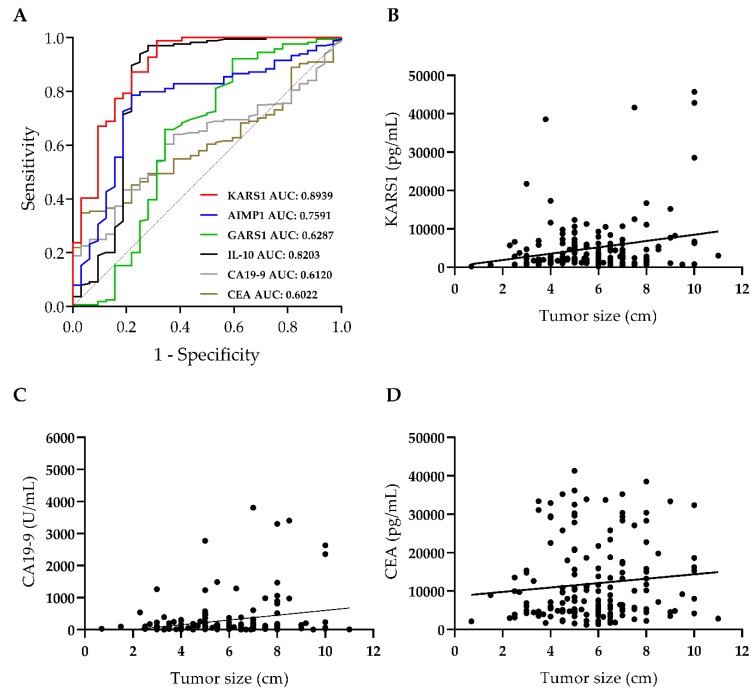
Diagnostic values of plasma KARS1 in CRC patients. Receiver operating characteristics (ROC) curves for Aminoacyl tRNA Synthetases (ARSs) and cancer biomarkers were analyzed. ROC curves of plasma KARS1, AIMP1, GARS1, IL-10, CA 19-9, and CEA (**A**) were used to differentiate CRC patients from healthy controls. Correlation of CRC plasma levels of proteins, including KARS1 (**B**), CA 19-9 (**C**), and CEA (**D**), with tumor size analyzed using Pearson correlation coefficient; AUC: Area under ROC curve.

Based on the initial findings, we narrowed down the novel biomarker candidates to KARS1, AIMP1, GARS1, and IL-10, and further evaluated their diagnostic potential. The same cohort results, previously determined by ELISA assay, were further analyzed by ROC analysis. Using ROC analysis, their sensitivity and specificity were determined and compared to the current biomarkers, CA19-9 and CEA (Table S2). ROC analysis showed that the diagnostic capability of plasma KARS1 (AUC: 0.8939, *p <* 0.0001) was highest followed by IL-10 (AUC: 0.8203, *p <* 0.0001), AIMP1 (AUC: 0.7591, *p <* 0.0001), and GARS1 (AUC: 0.6287, *p =* 0.0214). These findings showed that the sensitivity and specificity of plasma KARS1 was the highest for CRC samples. Next, the AUC values of plasma KARS1 were used to compare with those of known current biomarkers, CA 19-9 (AUC: 0.612, *p* = 0.0451) and CEA (AUC: 0.6022, *p* = 0.0676) (Figure 2A; Table S2). The results show that KARS1 and IL-10 had higher sensitivity and specificity than CEA and CA19-9 in CRC. Since the currently used CRC biomarkers are also found in other types of cancer, they are not specific for cancer segmentation. To determine the specificity of plasma KARS1 level in CRC, the levels of KARS1, CEA, and CA 19-9 were also measured by ELISA in the plasma of pancreatic cancer (PC) patients (*n* = 30) (Figure S1A–E). As previously reported, CEA and CA 19-9 showed significant differences in their plasma levels between PC and healthy controls, but not KARS1, implying the specificity of KARS1 toward CRC. The diagnostic value of plasma KARS1 levels in CRC was further analyzed using clinical characteristics. The Pearson correlation coefficient (r) was used to analyze the correlation between the tumor size and plasma level of each marker. Whereas plasma AIMP1 (*r* = −0.0506, *p* = 0.52), GARS1 (*r* = 0.0838, *p* = 0.286) and IL-10 (*r* = 0.0683, *p* = 0.385) did not show a meaningful r value, representing a noncorrelation with tumor size, the r value of plasma KARS1 (*r* = 0.2183, *p* = 0.005), CEA (*r* = 0.1084, *p* = 0.167) and CA 19-9 (*r* = 0.2322, *p* = 0.003) showed a positive correlation to tumor size (Figure 2B–D, Figure S2 and Table S3). Considering *p* value, only plasma KARS1 and CA 19-9 levels were statistically meaningful in the Pearson correlation coefficient analysis. Plasma KARS1 can be used as a diagnostic biomarker with the combined results of the ROC and Pearson correlation coefficient analysis. Plasma KARS1 can be used as a diagnostic biomarker by combining the results of the ROC and Pearson r analysis. Based on the plasma level and its correlation with the cancer burden in CRC, we found that the diagnostic potential of KARS1 was better than that of CEA and CA19-9.

### 3.3. Validation of Diagnostic Potential of Plasma KARS1 Using Colitis-Induced CRC Mouse Model

**Figure 3:**
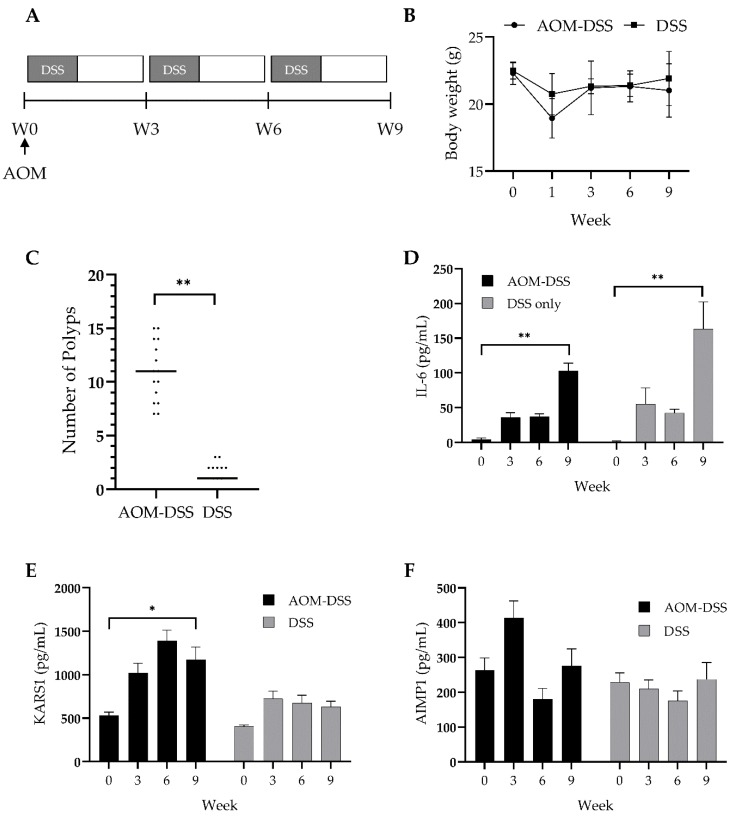
Plasma KARS1 level in colitis-induced colon cancer mouse model. (**A**) Schematic representation of azoxymethane/dextran sodium sulfate (AOM/DSS) treatment. (**B**) Body weight was monitored during the experiment for the AOM/DSS and DSS only treatment groups. (**C**) The number of polyps were measured. Every three weeks, interleukin 6 (IL-6) (**D**), KARS1 (**E**), and AIMP1 (**F**) levels were determined using ELISA from the plasma isolated from AOM/DSS and DSS mice. * *p* < 0.05, ** *p* < 0.0001.

As KARS1 is secreted during inflammation and CRC is highly associated with inflammation [28,29,30], plasma KARS1 was investigated using the colitis-induced CRC mouse model, azoxymethane (AOM)/dextran sodium sulfate (DSS) [31]. To compare plasma KARS1 levels in colitis and colitis-induced CRC, mice were divided into AOM/DSS and DSS only groups. Mice were intraperitoneally injected with PBS or AOM and were then given 2.5% DSS in their drinking water. After one week, the DSS was removed from the water for two weeks, which was considered one cycle. The mice were treated for a total of three cycles as shown in the schematics (Figure 3A). After seven days, the AOM/DSS and DSS only group had bloody stools and declining body weight. After two more cycles of DSS ingestion without AOM injection, the AOM/DSS group exhibited more severe bloody stool compared to the DSS only group. However, there was no significant difference in the body weight between the two groups (Figure 3B). After three cycles, the mice were euthanized to check for polyps in the colon. The number of colon polyps in the AOM/DSS group was greater than that of DSS only group (Figure 3C).

To investigate the cytokines and plasma KARS1 levels, plasma was collected every three weeks and analyzed with ELISA. IL-6, a well-known inflammatory cytokine in several inflammatory forms of cancer [32,33], was elevated with little difference between the AOM/DSS and DSS only groups (Figure 3D). In contrast, the plasma KARS1 level was highly increased in AOM/DSS group compared to DSS group, (Figure 3E), while there was no significant in AIMP1 levels (Figure 3F). These results show that plasma KARS1 is highly correlated to the carcinogenesis and presence of CRC rather than to only inflammation.

### 3.4. Validation of Monitoring Potential of Plasma KARS1 in CRC

Since plasma KARS1 level was correlated with tumor size of CRC, we investigated whether plasma KARS1 could be used as a monitoring biomarker. To validate the monitoring potential of plasma KARS1, plasma samples for 60 paired pre- and post-surgery CRC patients were collected (Table 2).

**Figure 4:**
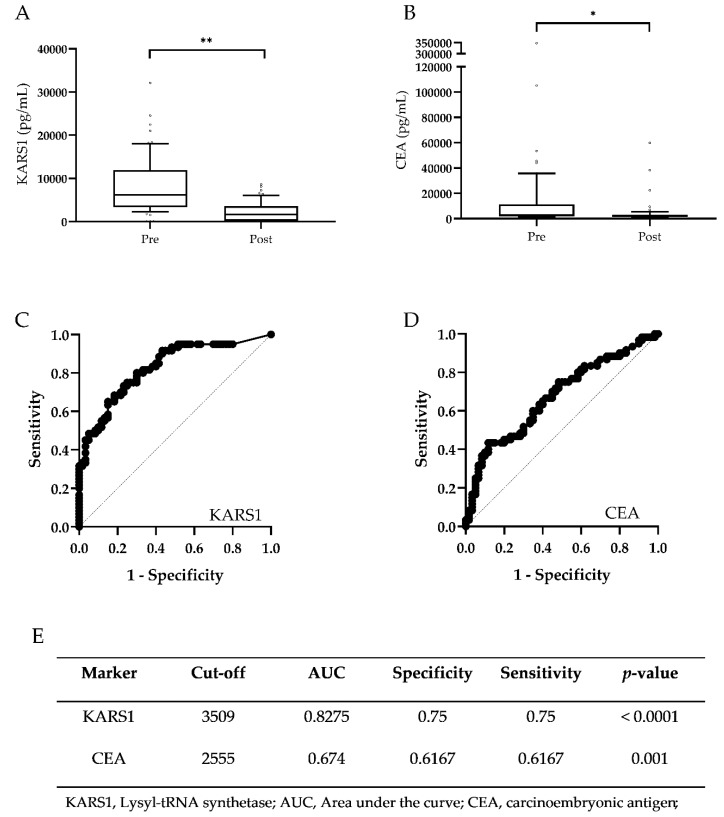
Monitoring values of plasma KARS1 in CRC. Levels of KARS1 (**A**) and CEA (**B**) in the plasma of paired pre- and post-surgery CRC patients (*n* = 60) were monitored and shown as box and whisker plots. ROC curves for KARS1 (**C**) and CEA (**D**) were used to differentiate post-surgery CRC from the pre-surgery CRC group. (**E**) Summary of ROC analysis, including the cutoff value, area under the ROC curve (AUC), specificity, sensitivity, and *p-*value. * *p* < 0.05, ** *p <* 0.0001.

To remove the pre-secreted plasma KARS1 from primary tumors, post-surgery CRC plasma was collected at five days after surgery. The plasma level of KARS1 and CEA was significantly decreased in post-surgery patients compared with pre-surgery patients (Figure 4A,B; Table S4). Plasma KARS1 (*p <* 0.0001) showed greater statistical difference than CEA (*p =* 0.0293) (Table S4). Further analysis was performed to check the monitoring potential for post-operative care with monitoring with plasma KARS1 levels in paired pre- and post-surgery samples using ROC analysis. This confirmed the significant monitoring value of plasma KARS1 levels (AUC: 0.8275, *p <* 0.0001), which was better than that of CEA (AUC: 0.6740, *p =* 0.001) (Figure 4C–E). These results further support that plasma KARS1 is correlated with CRC and has better monitoring capability than the current monitoring marker, CEA.

## 4. Discussion

The incidence of CRC is increasing worldwide, especially in early- and middle-aged people [2]. Although CRC can be diagnosed at an early stage using current diagnostics, the mortality rate has remained high due to issues with patient compliance for diagnostics, especially structural examination and recurrence [34]. CRC patients show 40% recurrence after surgery because remaining residues of the primary tumor may still exist, so post-operative care with screening is necessary for improving the survival rate [35]. Nevertheless, some patients do not comply with the structural diagnostic examination. Even if the clinician performs colonoscopy or CT scan as a follow-up, there is a chance the recurrence could go undetected. Therefore, there is a clinical need for a reliable and sensitive monitoring marker to detect the recurrence early, so that the clinicians can give proper treatment to help the survival of the patient. One method to overcome this is to use reliable blood-based CRC biomarkers. Although the established markers, CEA and CA19-9, were discovered several decades ago [36,37], there has been an increase in the false-positives for CA19-9 and CEA, especially for CEA levels after surgery [38,39,40]. Whereas other blood-based markers are promising diagnostic tools, they have rarely been recommended for cancer monitoring during and after surgery in international guidelines [41]. Due to this, physicians prefer using endoscopic or stool tests for screening and post-operation monitoring of CRC patients despite their inconvenience and cost [42]. Hence, physicians and patients alike would prefer a reliable diagnostic tool based on plasma biomarkers instead of the structural examination [43].

In our study, we showed that KARS1 is detected in the blood and can offer a more specific and precise diagnosis and monitoring than current CRC biomarkers. To our knowledge, we demonstrated the clinical use of KARS1 as a biomarker in CRC for the first time in this pilot study. Since KARS1 level can be monitored using plasma, this sampling method is more convenient for the patients compared to structural examination. Furthermore, the results show the induction and reduction of plasma KARS1 in AOM/DSS mice models and post-surgery CRC patients, respectively. Hence, KARS1 can be used as a potential diagnostic and monitoring biomarker for post-operative care and can contribute to improved management and follow-up treatments. Our data provides support for the clinical applications of plasma KARS1 levels for the detection of CRC, especially during the early stage and after surgery.

Our results, which showed that plasma KARS1 was higher in CRC patients compared to healthy individuals, can be used as a promising noninvasive diagnostic tool for clinicians and patients. Moreover, KARS1 is more sensitive and specific than currently used CRC biomarkers, such as CEA and CA19-9, to distinguish CRC patients from healthy control. These findings show that KARS1 is secreted into the blood from the tumors of CRC patients, and are consistent with our previous report that KARS1 is secreted from colon cancer cell lines. Since plasma KARS1 is highly correlated to primary tumor size of CRC patients and secreted KARS1 induces macrophage M2 polarization [24], it can be attributed to CRC tumorigenesis and metastasis, perhaps functionally associated with its macrophage M2 activity [44,45]. However, more mechanistic and clinical studies are needed to validate the etiological contribution of plasma KARS1 to CRC.

Although the mechanism behind high plasma KARS1 levels during the pathogenesis of CRC is not clear, it might have diagnostic potential for inflammatory bowel disease (IBD)-derived CRC. In AOM/DSS mice models, the plasma KARS1 level was similar between the control and DSS only treatment group. Plasma KARS1 was increased only in the AOM-DSS treatment group. Therefore, plasma KARS1 could also be used to detect IBD-derived CRC. In addition, specific patient cohort-related colorectal cancer, such as ulcerative colitis (UC) and Crohn’s disease (CD), are required to further establish the clinical significance of these findings.

## 5. Conclusions

Our data shows that the level of Plasma KARS1 reflects the tumor size in CRC patients and significantly associated with CRC. It was found to be more specific and sensitive than current CRC biomarkers used for diagnosis and monitoring. In addition, the level of KARS1 in CRC plasma was decreased post-surgery, which further strengthened the clinical relevance of plasma KARS1 in CRC. Based on these findings, we would like to expand our research for use in the clinical field.

## Figures and Tables

**Table 1:** Clinical features of healthy controls and CRC.

			Healthy	CRC
			No.	No.
Total(*n*)			32	164
Sex	Male(*n*)		20	93
	Female(*n*)		12	71
Age (years)			44.3 ± 9.27	60.2 ± 12.3
Tumor size (cm)			-	5.75 ± 1.89
Cancer Grade		I	-	14
		II	-	50
		III	-	50
		IV	-	50

CRC, colorectal cancer; No., Number.

**Table 2:** Clinical features of paired pre- and post-surgery CRC patients.

		CRC
		No.
Total(*n*)		60
Sex	Male(*n*)	44
	Female(*n*)	16
Age (years)		58.45 ± 10.63
Tumor size (cm)		1.88 ± 1.30
Cancer Grade	I	-
	II	3
	III	47
	IV	10

CRC, colorectal cancer; No., Number.

## Supplementary Materials

The following are available online at https://www.mdpi.com/2077-0383/9/2/533/s1, Table S1: Comparison of the plasma levels of ARSs and cancer biomarkers between healthy controls and CRC patients; Table S2: Statistical summary for ROC analysis of plasma proteins; Table S3: Statistical summary for Pearson correlation coefficient (Pearson r) of plasma proteins; Table S4: Comparison of the plasma level of KARS1 and CEA in paired pre- and post-surgery CRC patients; Figure S1: CRC specificity of plasma KARS1 as biomarker; Figure S2: Pearson correlation coefficient of AIMP1, GARS1 and IL-10.
