# Supplementary material for: Plasma Lysyl-tRNA Synthetase 1 (KARS1) as a Novel Diagnostic and Monitoring Biomarker for Colorectal Cancer
Source: J Clin Med. 2020 Feb 15;9(2):533. doi: 10.3390/jcm9020533 (PMC7073917; doi:10.3390/jcm9020533)
Supplement: Supplementary file 1 [file jcm-09-00533-s001.zip › Supplementary legend.docx]

**Plasma Lysyl-tRNA Synthetase 1 (KARS1) as a Novel Diagnostic and Monitoring Biomarker for Colorectal Cancer**

Ji Hun Suh^1,2§^, Min Chul Park^1§^, Peter C. Goughnour^1,2§^, Byung Soh Min^3^, Sang Bum Kim^1^, Woo Yong Lee^4,5^, Yong Beom Cho^4,5^, Jae Hee Cheon^6^, Kang Young Lee^3^, Do-Hyun Nam^5,7,8^ and Sunghoon Kim^1,2*^

**Supplementary figure legends**

**Supplementary Table S1. Comparison of the plasma levels of ARSs and cancer biomarkers between healthy controls and CRC patients.** Plasma levels were presented as mean ± SEM. Except for CA 19-9 (U/mL), pg/mL was used as the unit for concentration.

**Supplementary Table S2. Statistical summary for ROC analysis of plasma proteins**. Statistical values of plasma protein levels were summarized

**Supplementary Table S3. Statistical summary for Pearson correlation coefficient (Pearson r) of plasma proteins**. Statistical values of plasma protein levels were summarized

**Supplementary Table S4. Comparison of the plasma level of KARS1 and CEA in paired pre- and post-surgery CRC patients.** Values were presented as mean ± SEM. The unit used for concentration was pg/mL.

**Supplementary Figure S1. CRC specificity of plasma KARS1 as biomarker. (A)** Clinical features of pancreatic cancer (PC) patients. Plasma levels of KARS1 **(B)**, CEA **(C)** and CA-19-9 **(D)** were determined in PC patients and compared with the CRC as well as healthy group **(E)**. *P* values were analyzed using Mann-Whitney U test.

**Supplementary Figure S2. Pearson correlation coefficient of AIMP1, GARS1 and IL-10**. Correlation of the plasma protein levels in CRC patients, including AIMP1 **(A)**, GARS1 **(B)** and IL-10 **(C)**, with tumor size was analyzed by Pearson correlation coefficient.
