# Supplementary material for: Plasma Lysyl-tRNA Synthetase 1 (KARS1) as a Novel Diagnostic and Monitoring Biomarker for Colorectal Cancer
Source: J Clin Med. 2020 Feb 15;9(2):533. doi: 10.3390/jcm9020533 (PMC7073917; doi:10.3390/jcm9020533)
Supplement: Supplementary file 1 [file jcm-09-00533-s001.zip › supplementary TableS1.pdf]

# Supplementary Table S1

**Table S1. Comparison of the plasma levels of ARSs and cancer biomarkers between healthy controls and CRC patients**

| (pg/mL) | **Healthy** | **CRC** | ***p*-value** |
|---|---|---|---|
| AIMP1 | 1711±143.5 | 2600±68.32 | < 0.0001 |
| GARS1 | 561.2±137.3 | 535.5±39.08 | < 0.05 |
| HARS1 | 1574±290.1 | 1302±55.33 | 0.602 |
| KARS1 | 775.6±232.8 | 5007±561.1 | < 0.0001 |
| WARS1 | 2345±276.2 | 2683±127.1 | 0.2304 |
| TNF-$\alpha$ | 142.5±20.01 | 113.6±5.092 | 0.196 |
| IL-10 | 87.40±13.17 | 167.9±4.54 | < 0.0001 |
| CA 19-9 | 76.63±11.80 | 284.3±48.03 | < 0.05 |
| CEA | 6941±664.2 | 11939±785.8 | 0.0676 |

mean ± SEM, CA 19-9 unit: U/mL,;SEM, Standard error of the mean; CRC, Colorectal cancer; AIMP1, Aminoacyl tRNA synthetase-interacting multifunctional protein 1; GARS1, Glycyl-tRNA synthetase 1; HARS1, Histidyl-tRNA synthetase 1; KARS1, Lysyl-tRNA synthetase 1; WARS1, Tryptophanyl-tRNA synthetase 1; TNF, tumor necrosis factor; IL, Interleukin; CEA, Carcinoembryonic antigen; *p* values were calculated using the Mann-Whitney U test. * $p < 0.05$, ** $p < 0.0001$.
