# Supplementary material for: Plasma Lysyl-tRNA Synthetase 1 (KARS1) as a Novel Diagnostic and Monitoring Biomarker for Colorectal Cancer
Source: J Clin Med. 2020 Feb 15;9(2):533. doi: 10.3390/jcm9020533 (PMC7073917; doi:10.3390/jcm9020533)
Supplement: Supplementary file 1 [file jcm-09-00533-s001.zip › supplementary TableS2.pdf]

Supplementary Table S2

Table S2. Statistical summary for ROC analysis of plasma proteins.

Marker	Cutoff value	AUC	*p* value	Specificity	Sensitivity
KARS1	>1028 (pg/mL)	0.8939	< 0.0001	0.7813	0.8598
AIMP1	>1963 (pg/mL)	0.7591	< 0.0001	0.7813	0.7866
GARS1	>279.2 (pg/mL)	0.6287	0.0214	0.6250	0.6585
IL-10	>125.5 (pg/mL)	0.8203	< 0.0001	0.7813	0.8232
CA 19-9	>64.17 (U/mL)	0.6120	0.0451	0.6250	0.6402
CEA	>6407 (pg/mL)	0.6022	0.0676	0.5625	0.5610

AUC, Area under the curve; AIMP1, Aminoacyl tRNA synthetase-interacting multifunctional protein 1; GARS1, Glycyl-tRNA synthetase 1; KARS1, Lysyl-tRNA synthetase 1; IL, Interleukin; CEA, Carcinoembryonic antigen; Cutoff Value defined based on maximized the total value of sensitivity and specificity, ; *p* value, computes the SE differently, assuming that the area is really 0.5 (the null hypothesis) with two-tailed at prism.; value of specificity and sensitivity is value at cut-off.
