# Supplementary material for: Plasma Lysyl-tRNA Synthetase 1 (KARS1) as a Novel Diagnostic and Monitoring Biomarker for Colorectal Cancer
Source: J Clin Med. 2020 Feb 15;9(2):533. doi: 10.3390/jcm9020533 (PMC7073917; doi:10.3390/jcm9020533)
Supplement: Supplementary file 1 [file jcm-09-00533-s001.zip › supplementary TableS3.pdf]

Supplementary Table S3

Table S3. Statistical summary for Pearson correlation coefficient (Pearson r) of plasma proteins.

Marker	Pearson r	*p*- value
KARS1	0.2183	0.005
AIMP1	-0.0506	0.520
GARS1	0.0838	0.286
IL-10	0.0683	0.385
CA 19-9	0.2322	0.003
CEA	0.1084	0.167

AIMP1, Aminoacyl tRNA synthetase-interacting multifunctional protein 1; GARS1, Glycyl-tRNA synthetase 1; KARS1, Lysyl-tRNA synthetase 1; IL, Interleukin; CEA, Carcinoembryonic antigen; Each of Pearson r value was calculated based on comparison between tumor size and plasma marker level. *p* value computed two-tailed null hypothesis.
