# Supplementary material for: Plasma Lysyl-tRNA Synthetase 1 (KARS1) as a Novel Diagnostic and Monitoring Biomarker for Colorectal Cancer
Source: J Clin Med. 2020 Feb 15;9(2):533. doi: 10.3390/jcm9020533 (PMC7073917; doi:10.3390/jcm9020533)
Supplement: Supplementary file 1 [file jcm-09-00533-s001.zip › supplementary TableS4.pdf]

Supplementary Table 4

Table S4. Comparison of the plasma level of KARS1 and CEA in paired pre- and post-surgery CRC patients

(pg/mL)	**Pre**	**Post**	***p*-value**
KARS1	8224±861.4	2273±300	< 0.0001
CEA	16008±6066	4236±1181	0.0293

mean ± SEM; SEM, Standard error of the mean; KARS1, Lysyl-tRNA synthetase 1; CEA, Carcinoembryonic antigen, *p* values were calculated using Paired student's t-test (two-tailed) . * $p < 0.05$, ** $p < 0.0001$.
